# Trauma exposure, contextual stressors, and PTSD symptoms: patterns in racially and ethnically diverse, low-income postpartum women

**DOI:** 10.1017/S0033291724002915

**Published:** 2024-12

**Authors:** Yasmin B. Kofman, Joni Brown, Christine Dunkel Schetter, Jennifer A. Sumner

**Affiliations:** Department of Psychology, University of California Los Angeles, Los Angeles, CA, USA

**Keywords:** contextual stress, latent class analysis, posttraumatic stress disorder, trauma

## Abstract

**Background:**

Racial, ethnic, and socioeconomic disparities persist in posttraumatic stress disorder (PTSD), which are partly attributed to minoritized women being trauma-exposed, while also contending with harmful contextual stressors. However, few have used analytic strategies that capture the interplay of these experiences and their relation to PTSD. The current study used a person-centered statistical approach to examine heterogeneity in trauma and contextual stress exposure, and their associations with PTSD and underlying symptom dimensions, in a diverse sample of low-income postpartum women.

**Methods:**

Using a community-based sample of Black, Hispanic/Latina, and White postpartum women recruited from five U.S. regions (*n* = 1577), a latent class analysis generated profiles of past-year exposure to traumatic events and contextual stress at one month postpartum. Regression analyses then examined associations between class membership and PTSD symptom severity at six months postpartum as a function of race/ethnicity.

**Results:**

A four-class solution best fit the data, yielding High Contextual Stress, Injury/Illness, Violence Exposure, and Low Trauma/Contextual Stress classes. Compared to the Low Trauma/Contextual Stress class, membership in any of the other classes was associated with greater symptom severity across nearly all PTSD symptom dimensions (all *ps* < 0.05). Additionally, constellations of exposures were differentially linked to total PTSD symptom severity, reexperiencing, and numbing PTSD symptoms across racial/ethnic groups (*ps* < 0.05).

**Conclusions:**

A person-centered approach to trauma and contextual stress exposure can capture heterogeneity of experiences in diverse, low-income women. Moreover, racially/ethnically patterned links between traumatic or stressful exposures and PTSD symptom dimensions have implications for screening and intervention in the perinatal period.

## Introduction

In the United States, racial, ethnic, and socioeconomic disparities persist in the prevalence and development of posttraumatic stress disorder (PTSD). Black Americans in particular have been found to have higher lifetime prevalence of PTSD and higher conditional risk (i.e. the percentage of those who develop PTSD after trauma exposure) compared to other racial groups (Alcántara, Casement, & Lewis-Fernandez, 2013; Roberts, Gilman, Breslau, Breslau, & Koenen, 2011). Research has also consistently documented differences in PTSD for Latinos, with conditional risk for PTSD at times higher than that of non-Latino Black individuals (Alcántara et al., 2013; Hall-Clark, Sawyer, Golik, & Asnaani, 2016). These disparities are significant public health concerns with profound societal costs, as PTSD impairs day-to-day functioning (Jellestad, Vital, Malamud, Taeymans, & Mueller-Pfeiffer, 2021), is associated with elevated risk for developing other mental health disorders (Goldstein et al., 2016), and has increasingly been tied to chronic physical health problems (Schnurr, 2015).

These observed inequalities in PTSD are partly attributed to disproportionate rates of trauma exposure and the distinctive types of trauma experienced by minoritized groups. For example, Black, Hispanic, and Native Americans are more likely to experience interpersonal violence, compared to White Americans, who are more likely to experience non-interpersonal and accidental traumas (Hall-Clark et al., 2016; Spoont & McClendon, 2020). While trauma exposure alone can trigger the development of PTSD, traumatic events rarely occur in a vacuum. There is growing recognition of the context dependent nature of traumatic experiences, highlighting the importance of environmental and social factors often overlooked in PTSD research. Integration of frameworks such as the Social Determinants of Health (SDoH; Comission of Social Determinants of Health, 2008) emphasizes the importance of broader environmental and social factors in health and health disparities, (Alegría, NeMoyer, Falgàs Bagué, Wang, & Alvarez, 2018; Allen, Balfour, Bell, & Marmot, 2014). Contextual stressors within SDoH domains, such as poverty, neighborhood crime, and discrimination, not only increase likelihood of trauma exposure but also compound the risk for the development and maintenance of PTSD (Holder, Mehlman, Delgado, & Maguen, 2022).

Due to multiple intersections of gender, race, ethnicity, and socioeconomic status, low-income, minoritized women are particularly prone to the hazardous interplay of trauma and contextual stress exposure. For example, Black and non-White Hispanic women are more likely to experience poverty-related contextual stressors, which have been linked to greater PTSD symptom severity and likelihood of PTSD diagnosis (Holmes et al., 2021; Whittle et al., 2019). Women from impoverished communities are also more likely to experience traumatic events, such as car accidents, witnessing violence, or being victims of assault (Hong & Burnett-Zeigler, 2017; Ravi, Powers, Rothbaum, Stevens, & Michopoulos, 2023b). Similarly, minoritized women are more likely to experience racial or ethnic discrimination, which has been linked to heightened risk for psychological distress and PTSD, along with elevated PTSD symptomatology (Mekawi et al., 2021; Ravi et al., 2023a). Discrimination has also been linked to greater trauma exposure in the form of interpersonal violence (e.g. physical and sexual violence; Raj et al., 2023).

The perinatal period, which can extend from pregnancy through one year postpartum, is a stress-sensitive juncture when heightened experiences of contextual stress and trauma may be particularly impactful (O'Hara & Wisner, 2014). During this time, physiological, psychological, and social changes occur that can exacerbate pre-existing mental health conditions or symptoms and introduce new ones (Frieder, Dunlop, Culpepper, & Bernstein, 2008; Meltzer-Brody et al., 2018; Yildiz, Ayers, & Phillips, 2017). Certain contextual stressors and traumatic exposures may also be more salient during this time. For instance, increased interaction with the healthcare system can introduce an additional layer of discrimination. Black women, for example, have reported biased and unfair treatment by healthcare professionals during prenatal care visits (Salm Ward, Mazul, Ngui, Bridgewater, & Harley, 2013). In terms of trauma exposure, low-income women face disproportionately high levels of IPV exposure, with documented prevalence rates as high as 50% (Alhusen, Ray, Sharps, & Bullock, 2015). These intersecting experiences heighten vulnerability to posttraumatic psychopathology, with negative implications for maternal-infant health (Wisner, Murphy, & Thomas, 2024). Yet, to the best of our knowledge, research investigating this complex interplay in relation to PTSD outcomes in marginalized women during the perinatal period has not been explored.

In the current study, we used a person-centered statistical approach, latent class analysis (LCA), to examine patterns of trauma and contextual stressors and their associations with PTSD symptoms in a diverse group of low-income postpartum women. We also investigated how identified latent class profiles were associated with underlying PTSD symptom dimensions given the heterogeneous nature of PTSD symptoms and the distinct maternal health and functioning correlates associated with specific symptom dimensions (Galatzer-Levy & Bryant, 2013; Thomas, Cleveland, Pietrzak, Dunkel Schetter, & Sumner, 2021b). Prior research with this sample has indicated disparities in manifestations of these symptom dimensions, with Black and Hispanic/Latina women endorsing more fear-related symptoms of PTSD, including symptoms of active avoidance and reexperiencing of trauma reminders, and White women endorsing dysphoric arousal symptoms (Thomas, Carter, Dunkell Schetter, & Sumner, 2021a). The current study expands on that work by investigating how underlying patterning of trauma and contextual stress exposure may relate to PTSD symptoms differentially by race/ethnicity. This work addresses an important gap in understanding risk factors and can inform tailoring of interventions to address the needs of diverse populations in the perinatal context.

## Method

### Participants and procedure

Data were collected by the Community Child Health Network (CCHN), a multi-site, community-based participatory research network that conducted a study on stress, resilience, and maternal–child health outcomes in several low-income communities (see Ramey et al., 2015 for details). Approximately 2500 Black, Hispanic/Latina, and White mothers were recruited from five sites (Baltimore, MD, eastern North Carolina, Lake County, IL, Los Angeles, CA, and Washington, DC) in hospitals at the time of birth, except one site that recruited prenatally (NC). In-home interviews were conducted in English or Spanish at 1, 6, 12, 18, and 24 months after birth. Informed consent was obtained at recruitment, and this research was approved by the Institutional Review Boards at all institutions involved. Our analytic sample consisted of 1577 mothers interviewed at both 1 and 6 months postpartum who had complete data on key variables.

### Measures

#### Trauma exposure

At one month postpartum, past-year trauma exposure for self or close others was assessed using a shortened version of the 24-item Life Events Checklist (Dominguez, Schetter, Mancuso, Rini, & Hobel, 2005). As in prior research (Thomas et al., 2021a), we identified seven potentially traumatic events (PTEs) that aligned with the DSM-IV-TR's Criterion A for trauma: (1) serious injury, illness, or hospitalization (self or close other); (2) being mugged or personally attacked; (3) death of close other(s); (4) serious motor vehicle accident; (5) being threatened with physical harm; (6) being robbed or burglarized; (7) being a victim of a violent crime. Additionally, past-year IPV exposure was captured using a modified HITS Scale (Sherin, Sinacore, Li, Zitter, & Shakil, 1998; Velonis et al., 2017), including the original four items and an additional item on having actions or activities restricted by a partner/spouse. Responses were rated from 1 (*never*) to 5 (*frequently*). For the current study, only experiences perpetrated by a partner/spouse were considered. A binary indicator was created to indicate presence/absence of any past-year IPV.

#### Contextual stressors

Three major contextual stressors within SDoH domains were assessed at 1 month postpartum. First, *neighborhood safety* was assessed with one item about crime frequency in participants' neighborhood (Coulton, Korbin, & Su, 1996): ‘How often are there problems with muggings, burglaries, assaults or anything else like that in your neighborhood, or the area where you live?’. Responses of ‘very often’ or ‘fairly often’ were coded as exposure to unsafe neighborhoods, while responses of ‘not too often’, ‘hardly ever’, or ‘never’ were coded as unexposed. Next, *household food insecurity* in the last 12 months was assessed with two items that queried whether food didn't last and whether funds to purchase more food were lacking (Bickel, Nord, Price, Hamilton, & Cook, 2000). Women endorsing any challenges for either item were coded as food insecure. Finally, *discrimination* attributed to ancestry/national origins, race, skin color, or language and accent was assessed with the 9-item Everyday Discrimination Scale (Williams, Yu, Jackson, & Anderson, 1997). Experiencing any of the items ‘a few times a year’ or more was coded as being exposed to discrimination, while ‘never’ or ‘less than once a year’ across all items was coded as unexposed.

#### PTSD symptoms

The 17-item PTSD Checklist-Civilian Version (PCL-C; Blanchard, Jones-Alexander, Buckley, & Forneris, 1996) was used to assess past-month PTSD symptoms at 6 months postpartum. Participants indicated the extent to which they were bothered by each of the DSM-IV PTSD symptoms in the past month on a 1 (*not at all*) to 5 (*extremely*) scale. A total PTSD symptom severity score was derived by summing item responses (Cronbach's *α* = 0.91). We also calculated symptom dimension scores for the five-factor dysphoric arousal model (Armour, Műllerová, & Elhai, 2016; Elhai et al., 2011), which has received extensive support as a dimensional model of PTSD (Armour et al., 2016) and was identified as the best-fitting model of PTSD in postpartum women in CCHN (Thomas et al., 2021b). Items were scored to reflect the following dimensions: (1) reexperiencing (e.g. intrusive thoughts; Cronbach's *α* = 0.84); (2) avoidance (e.g. avoidance of reminders; Cronbach's *α* = 0.72); (3) numbing (e.g. loss of interest; Cronbach's *α* = 0.78); (4) dysphoric arousal (e.g. difficulty sleeping or concentrating; Cronbach's *α* = 0.75); (5) anxious arousal (e.g. overly alert; Cronbach's *α* = 0.48).

### Statistical analyses

Descriptive statistics were reported as frequency counts and percentages for categorical variables and as means and standard deviations (SDs) for continuous variables. Group comparisons for all study variables by race/ethnicity were conducted using chi square tests or one-way analyses of variance.

LCA identified groups of individuals with similar trauma and contextual stress profiles. This finite mixture model sorted respondents into mutually exclusive groups based on the identified patterns (Collins & Lanza, 2009). Model selection was based on comparing several fit indices – Akaike's information criterion (AIC; Akaike, 1987), Bayesian information criterion (BIC; Schwarz, 1978), sample-size adjusted BIC (aBIC), and approximate Weight of Evidence Criterion (AWE) – where lower values indicated superior fit. Likelihood-based tests, including the Vuong Lo-Mendell-Rubin adjusted likelihood ratio test (VLMR-LRT) and the bootstrapped likelihood ratio test (BLRT), assessed whether increasing the number of classes lead to improvement in model fit. After class enumeration, average posterior probability (AvePP_k_) and entropy were used to assess classification accuracy and precision. Entropy ranges from 0 to 1; values >0.80 are regarded as reflecting high classification precision (Nylund-Gibson et al., 2023b). Posterior probabilities were then used to assign each respondent to a latent class. LCA models were estimated using Mplus (Muthén & Muthen, 2017).

Categorical variables of class membership and race/ethnicity were dummy coded to allow for comparisons of each category to a reference category. Separate regression analyses were conducted to examine the relationship between class membership, race/ethnicity, and PTSD symptoms (both total severity and symptom dimensions). Next, class ×  race/ethnicity interaction terms were included in regression models. Based on the omnibus test of the overall class ×  race/ethnicity interaction, individual class ×  race/ethnicity interaction coefficients were examined. Within-class contrasts assessed symptom severity differences within class by race/ethnicity. All regression models adjusted for site, education, poverty level, and age, which were collected at enrollment via self-report. Regression analyses were performed using Stata 17 (StataCorp, 2023).

## Results

### Participant characteristics

Participant characteristics are presented in Table 1. Women in the study sample were 26 years old on average, and Black and Hispanic/Latina women were slightly younger than White women. Most women (70%) were 100% below the federal poverty level. The largest proportion of women were high school educated (42%), and White women had higher levels of education than Black and Hispanic/Latina women, with 45% having a 4-year degree compared to 7% of Black and 5% of Hispanic/Latina women.

**Table 1.** Sociodemographics and distribution of main study variables by race/ethnicity (*n* = 1577)

Variable	All (*n* = 1577)	Black (*n* = 850)	White (*n* = 374)	Hispanic/Latina (*n* = 353)	*p*
Age	25.80 (5.72)	24.21 (4.98)	29.61 (5.91)	25.58 (5.31)	**<0.001**
Study site					**<0.001**
Baltimore	354 (22.45)	289 (34.00)	65 (17.38)	0 (0)	
Chicago	404 (25.62)	61 (7.18)	154 (41.18)	189 (53.54)	
Los Angeles	182 (10.02)	58 (6.82)	41 (10.96)	83 (23.51)	
North Carolina	326 (20.67)	222 (26.12)	100 (26.74)	4 (1.13)	
Washington, D.C.	311 (19.72)	220 (25.88)	14 (3.74)	77 (21.81)	
Poverty					**<0.001**
≤100% FPL	679 (43.06)	456 (53.65)	81 (21.66)	142 (40.23)	
100–200% FPL	430 (27.27)	208 (24.47)	64 (17.11)	158 (44.76)	
>200% FPL	468 (29.68)	186 (21.88)	229 (61.23)	53 (15.01)	
Education					**<0.001**
Less than high school	280 (17.76)	135 (15.88)	20 (5.35)	125 (35.41)	
High school, GED, certificate	669 (42.42)	417 (49.06)	97 (25.94)	155 (43.91)	
Some college	373 (23.65)	235 (27.65)	89 (23.80)	49 (13.88)	
4-year degree or higher	241 (15.28)	57 (6.71)	167 (44.65)	17 (4.82)	
Other/no information	14 (0.89)	6 (0.71)	1 (0.27)	7 (1.98)	
Trauma exposures					
Serious illness or injury	444 (28.15)	242 (28.47)	112 (30.20)	90 (25.50)	0.39
Mugged or personally attacked	109 (6.91)	67 (7.88)	13 (3.48)	29 (8.22)	**0.01**
Death of close other	437 (27.71)	272 (32.00)	86 (22.99)	79 (22.39)	**<0.001**
Motor vehicle accident	73 (4.63)	47 (5.54)	13 (3.48)	13 (3.69)	0.18
Threatened with physical harm	123 (7.80)	88 (10.35)	121 (5.61)	14 (3.98)	**<0.001**
Robbed or burglarized	75 (4.76)	38 (4.47)	24 (6.42)	13 (3.68)	0.19
Victim of violent crime	36 (2.29)	29 (3.42)	4 (1.07)	3 (0.85)	**0.005**
IPV	477 (35.33)	238 (33.95)	128 (39.26)	111 (34.37)	0.23
Contextual stressors					
Unsafe neighborhood	186 (11.94)	114 (13.60)	36 (9.68)	36 (10.34)	0.09
Food insecurity	340 (21.60)	219 (25.86)	43 (11.50)	78 (22.10)	**<0.001**
Everyday discrimination	579 (37.21)	348 (41.73)	61 (16.40)	170 (48.57)	**<0.001**
Number of traumas/stressors	1.83 (1.45)	2.00 (1.54)	1.44 (1.23)	1.80 (1.35)	**<0.001**
PTSD symptom severity	26.56 (10.09)	27.43 (11.03)	24.89 (8.27)	26.21 (9.22)	**<0.001**
Reexperiencing	7.80 (3.52)	8.18 (3.82)	7.08 (2.90)	7.64 (3.21)	**<0.001**
Avoidance	3.23 (1.79)	3.50 (1.97)	2.81 (1.41)	3.05 (1.59)	**<0.001**
Numbing	7.18 (3.09)	7.31 (3.36)	6.62 (2.47)	7.26 (2.93)	**<0.001**
Dysphoric arousal	5.00 (2.42)	4.92 (2.58)	5.40 (2.25)	4.78 (2.12)	**<0.001**
Anxious arousal	3.34 (1.56)	3.43 (1.84)	2.99 (1.44)	3.48 (1.56)	**<0.001**

FPL, federal poverty level; IPV, intimate partner violence; PTSD, posttraumatic stress disorder.

*Note*: *N* (%) for categorical variables; *M* (s.d.) for continuous variables. Significant *p* values ≤ 0.05 in **bold**.

Distributions of past-year PTEs and contextual stressors in the full sample and by race/ethnicity are also presented in Table 1. The most commonly endorsed PTE was IPV (35%), with no significant differences across race/ethnicity. However, group differences emerged for other PTEs. For example, Black and Hispanic/Latina women had higher instances of being mugged or personally attacked in the past year (both 8%) compared to White women (3%). Black women also had higher instances of death of a close other (32%), threats of physical harm (10%), and violent crime victimization (29%) than White and Hispanic/Latina women. In terms of past-year contextual stressors, experiences of everyday discrimination were quite prevalent among women in this sample (37%), with Black (42%) and Hispanic/Latina women (49%) having higher levels of discrimination than White women (22%). Food insecurity was also more prevalent in Black (26%) and Hispanic/Latina women (22%) than in White women (12%). Overall, Black women experienced the most total types of PTEs and contextual stressors (*M* = 2.00, s.d. = 1.54). PTSD symptoms are also presented in Table 2. Black and Hispanic/Latina women had the highest levels of symptom severity for total PTSD symptoms and across all dimensions except for dysphoric arousal.

**Table 2.** Associations between class membership, race/ethnicity, and PTSD symptom dimensions

	Total PTSD				Reexperiencing				Avoidance				Numbing				Dysphoric Arousal				Anxious Arousal			
	*F* (16, 1560) = 11.50, *p* < 0.001				*F* (16, 1560) = 11.00, *p* < 0.001				*F* (16, 1560) = 9.40, *p* < 0.001				*F* (16, 1560) = 9.08, *p* < 0.001				*F* (16, 1560) = 6.27, *p* < 0.001				*F* (16, 1560) = 9.40, *p* < 0.001			
Table 2a	*b*	s.e.	*β*	*p*	*b*	s.e.	*β*	*p*	*b*	s.e.	*β*	*p*	*b*	s.e.	*β*	*p*	*b*	s.e.	*β*	*p*	*b*	s.e.	*β*	*p*
Class																								
High Contextual stress	4.16	0.68	5.50	**<0**.**001**	1.31	0.24	0.14	**<0.001**	0.55	0.12	0.11	**<0.001**	1.27	0.21	0.15	**<0.001**	0.79	0.17	0.12	**<0.001**	0.25	0.12	0.05	**0.04**
Injury/Illness	3.43	0.61	4.62	**<0.001**	1.25	0.21	0.15	**<0.001**	0.33	0.11	0.08	**0.002**	0.67	0.19	0.09	**<0.001**	0.81	0.15	0.14	**<0.001**	0.37	0.11	0.09	**<0.001**
Violence Exposure	6.27	1.48	9.17	**<0.001**	2.36	0.52	0.11	**<0.001**	0.85	0.27	0.08	**0.001**	1.18	0.46	0.06	**0.01**	1.28	0.36	0.09	**<0.001**	0.62	0.26	0.06	**0.07**
Low Trauma/Contextual Stress (ref)	---	---	---	---	---	---	---	---	---	---	---	---	---	---	---	---	---	---	---	---	---	---	---	---
Race/Ethnicity																								
Black	−0.65	0.73	0.78	0.37	0.04	0.26	0.01	0.88	0.21	0.13	0.06	0.10	−0.10	0.23	−0.02	0.66	−0.85	0.18	−0.17	**<0.001**	0.04	0.13	0.01	0.74
Hispanic/Latina	−1.09	0.87	0.63	0.21	−0.28	0.31	−0.03	0.36	−0.20	0.16	−0.05	0.21	0.07	0.27	0.01	0.80	−0.92	0.22	−0.16	**<0.001**	0.24	0.15	0.12	0.12
White (ref)	---	---	---	---	---	---	---	---	---	---	---	---	---	---	---	---	---	---	---	---	---	---	---	---
	Total PTSD				Reexperiencing				Avoidance				Numbing				Dysphoric Arousal				Anxious Arousal			
Table 2b	*F* (16, 1560) = 11.50, *p* < 0.001				*F* (16, 1560) = 11.00, *p* < 0.001				*F* (16, 1560) = 9.40, *p* < 0.001				*F* (16, 1560) = 9.08, *p* < 0.001				*F* (16, 1560) = 6.27, *p* < 0.001				*F* (16, 1560) = 9.40, *p* < 0.001			
	*b*	s.e.	*β*	*p*	*b*	s.e.	*β*	*p*	*b*	s.e.	*β*	*p*	*b*	s.e.	*β*	*p*	*b*	s.e.	*β*	*p*	*b*	s.e.	*β*	*p*
Class																								
High Contextual Stress	4.16	0.68	5.50	**<0**.**001**	1.31	0.24	0.14	**<0.001**	0.55	0.12	0.11	**<0.001**	1.27	0.21	0.15	**<0.001**	0.79	0.17	0.12	**<0.001**	0.25	0.12	0.05	**0.04**
Injury/Illness	3.43	0.61	4.62	**<0.001**	1.25	0.21	0.15	**<0.001**	0.33	0.11	0.08	**0.002**	0.67	0.19	0.09	**<0.001**	0.81	0.15	0.14	**<0.001**	0.37	0.11	0.09	**<0.001**
Violence Exposure	6.27	1.48	9.17	**<0.001**	2.36	0.52	0.11	**<0.001**	0.85	0.27	0.08	**0.001**	1.18	0.46	0.06	**0.01**	1.28	0.36	0.09	**<0.001**	0.62	0.26	0.06	*0.07*
Low Trauma/Contextual Stress (ref)	---	---	---	---	---	---	---	---	---	---	---	---	---	---	---	---	---	---	---	---	---	---	---	---
Race/ethnicity																								
Black	0.44	0.77	0.02	0.57	0.32	0.27	0.05	0.24	0.41	0.14	0.11	**0.003**	−0.17	0.24	−0.03	0.48	0.08	0.19	0.02	0.69	−0.20	0.13	−0.06	0.14
White	−1.09	0.87	0.63	0.21	−0.28	0.31	−0.03	0.36	−0.20	0.16	−0.05	0.21	0.07	0.27	0.01	0.80	−0.92	0.22	−0.16	**<0.001**	0.24	0.15	0.12	0.12
Hispanic/Latina (ref)	---	---	---	---	---	---	---	---	---	---	---	---	---	---	---	---	---	---	---	---	---	---	---	---

*Note*: All models adjusted for mother's age, education, region, and poverty level. In Table 2a, the Low Trauma/Contextual Stress class and White race/ethnicity are specified as reference groups. In Table 2b, the Low Trauma/Contextual Stress class and Hispanic/Latina race/ethnicity are specified as reference groups. Significant *p* values ≤ 0.05 in **bold**. *p* values ≤ 0.10 are italicized, although these values did not reach statistical significance.

### Latent class model selection and results

**Figure 1.**
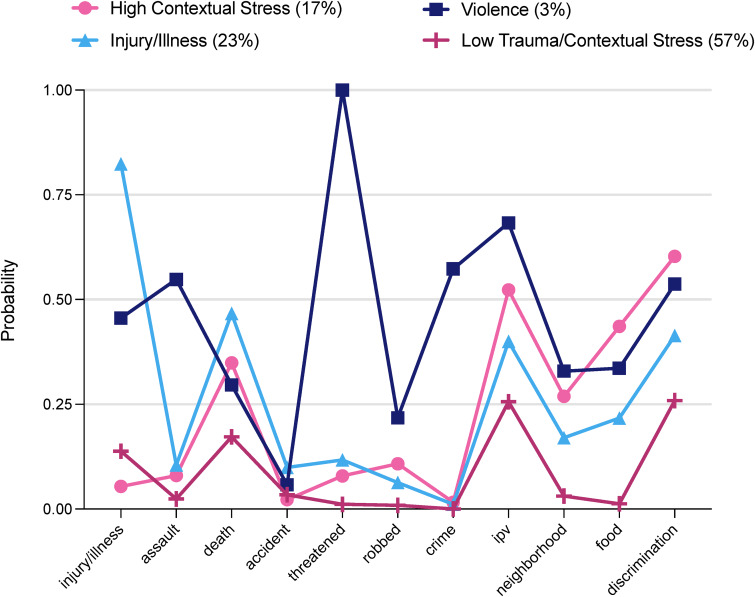
Item response probability plot for trauma and contextual stress exposure. Injury/illness = serious injury or illness; assault = mugged or personally attacked; death = death of a close other; accident = motor vehicle accident; threatened = threatened with physical harm; robbed = robbed or burglarized; crime = victim of violent crime; IPV = intimate partner violence; neighborhood = unsafe neighborhood; food = food insecurity; discrimination = everyday discrimination.

As commonly seen in LCA (Nylund-Gibson et al., 2023a; Nylund-Gibson & Choi, 2018), there was some disagreement across fit indices. BIC, aBIC, and CIAC were lowest for the 2- and 3-class models, indicating superior fit over models with four or more classes (online Supplementary Table S1). However, the difference in aBIC from the 3- to 4-class model was minimal. Likelihood-based tests indicated improvement in model fit up to the 5-class model (BLRT) and 2-class model (VLMR); taking into consideration that BLRT has been shown to be more robust and reliable compared to VLMR (e.g. Nylund, Asparouhov, & Muthén, 2007; Tein, Coxe, & Cham, 2013). Model classification proportions and accuracy (online Supplementary Table S2) revealed one class with less than 3% of the sample in all models except the 2-class model; however, methodological guidelines have suggested that if overall sample size is sufficiently large (*N* ≈ 300–1000), enumerating small classes may be stable and justifiable, especially with supporting theoretical and substantive factors (Nylund-Gibson et al., 2023a; Nylund-Gibson & Choi, 2018). Upon closer inspection, the smallest class across models exhibited a distinctive pattern of item endorsement for violent exposures (i.e. assault, being threatened, IPV, violent crime; Figure 1). Average posterior probability of classification values was satisfactory (near or above 0.7) for all models, indicating adequate classification precision. Entropy values ranged from below the recommended cut-off of 0.80 (0.57–0.63), suggesting some overlap across classes. Class interpretability slightly improved in the 4-class *v.* the 3-class model. The additional class in the 4-class model provided a distinctive pattern of item endorsement that was missed using the 3-class model, which included high endorsement for serious injury, illness, or hospitalization. Adding a 5th class did not meaningfully add to class interpretation. Considering these factors together, the 4-class model was selected as the best fit for the data.

The item response probability plot for the 4-class LCA was used to interpret and label latent classes for women (Fig. 1). The first class was characterized by higher levels of contextual stressors (e.g. food insecurity, everyday discrimination), but not necessarily PTEs, relative to the other classes. This class (labeled *High Contextual Stress*) was composed of 17% of the sample (*n* = 265). The second class included 23% of the sample (*n* = 353) and was characterized by high item response probabilities for serious illness or injury (*Illness*/*Injury* class). The third class included 3% of women (*n* = 45) and was characterized by high levels of violence, particularly threats of physical harm and IPV, along with elevated levels of being a victim of a serious crime (*Violence Exposure* class). Finally, the fourth class included most of the sample (57%; *n* = 914) and was characterized by lower item response probabilities across PTEs and contextual stressors (*Low Trauma & Contextual Stress* class).

Across classes, significant associations with age, site, poverty, education, and race were found (online Supplementary Table S3). Women in the *Low Trauma & Contextual Stress* class were older on average compared to the other classes. About half of women in the *High Contextual Stress*, *Injury*/*Illness*, and *Violence Exposure* classes were less than 100% of the federal poverty line, while in the *Low Trauma & Contextual Stress* class, women were more evenly dispersed across federal poverty levels. Notably, Black women were overrepresented in the *Violence Exposure* class.

### Associations between class membership, race/ethnicity, and PTSD symptoms

The first set of regression results (Table 2) examined the main effects of race/ethnicity and class membership with PTSD symptoms. Using the *Low Trauma & Contextual Stress* class as the reference class, significant associations were consistently found, such that women in the *High Contextual Stress*, *Injury*/*Illness*, and *Violence Exposure* classes had greater symptom severity for total PTSD symptoms and all symptom dimensions (all *ps* < 0.05, except for *Violence Exposure* and anxious arousal, which approached, but did not reach, the level of statistical significance [*p* = 0.07]). To examine the main effect of race, we first examined White women as the reference group (Table 2a), then reconducted analyses with Hispanic/Latina women as the reference group (Table 2b) to allow comparisons between all races/ethnicities. Significant associations between race/ethnicity and PTSD symptoms were only found for certain PTSD dimensions. Specifically, Black women had higher avoidance symptom severity compared to Hispanic/Latina women (*b* = 0.41, *p* = 0.003), and both Black (*b* = −0.85, *p* < 0.001) and Hispanic/Latina women (*b* = −0.92, *p* < 0.001) had lower dysphoric arousal symptom severity compared to White women. No other significant associations were found for race/ethnicity.

**Figure 2.**
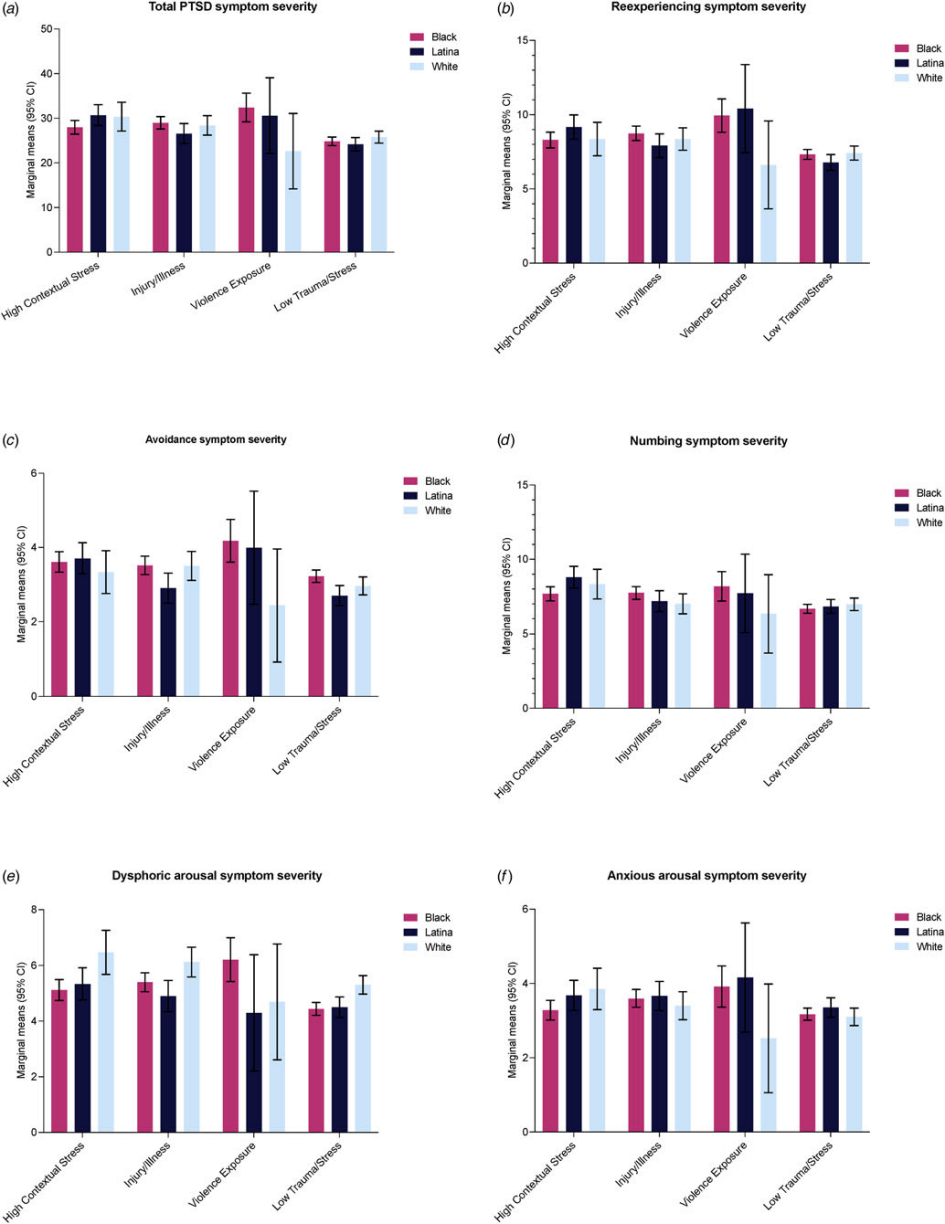
Class membership × race/ethnicity interaction plots.

In the next set of regressions, the class × race/ethnicity interaction was included in analyses (see Table 3 for interaction results with White race/ethnicity as reference group; see online Supplementary Table S4 for interaction results with Hispanic/Latina race/ethnicity as reference group). Significant interactions emerged for total PTSD symptom severity, reexperiencing, and numbing, as identified by omnibus tests (i.e. the difference in the differences of the predicted values), and these were interpreted further. For total PTSD, there was a greater difference in scores between Black and White women in the *Violence Exposure* class than in the *Low Trauma & Contextual* Stress class (*b* = 10.73, *p* = 0.02). Additionally, there was a greater difference in scores between Black and Hispanic/Latina women in the *High Contextual Stress* class than in the *Low Trauma & Contextual* Stress class (*b* = −3.37, *p* = 0.04). We further interpreted results by examining within-class contrasts (see online Supplementary Table S5 for contrasts of marginal linear predictions and Fig. 2 for plots). In the *Violence Exposure* class, Black women had higher total PTSD symptom severity than White women. Black women within the *Injury*/*Illness* class had higher total PTSD symptom severity than Hispanic/Latina women; this difference approached, but did not reach, the level of statistical significance. Similarly, Hispanic/Latina women within the *High Contextual Stress* class had greater total PTSD symptom severity than Black women.

**Table 3.** Class membership × race/ethnicity interactions and associations with PTSD symptom dimensions

	Total PTSD				Reexperiencing				Avoidance				Numbing				Dysphoric arousal				Anxious Arousal			
	*F* (22, 1554) = 9.02, *p* < 0.001				*F* (22, 1554) = 8.67, *p* < 0.001				*F* (22, 1554) = 7.36, *p* < 0.001				*F* (22, 1554) = 7.27, *p* < 0.001				*F* (22, 1554) = 5.02, *p* < 0.001				*F* (22, 1554) = 5.07, *p* < 0.001			
	*b*	s.e.	*β*	*p*	*b*	s.e.	*β*	*p*	*b*	s.e.	*β*	*p*	*b*	s.e.	*β*	*p*	*b*	s.e.	*β*	*p*	*b*	s.e.	*β*	*p*
Class																								
High stress	4.60	1.75	0.17	**0.01**	0.95	0.61	0.10	0.12	0.37	0.31	0.08	0.24	1.36	0.54	0.16	**0.01**	1.17	0.43	0.18	**0.01**	0.75	0.30	0.17	**0.01**
Injury and illness	2.64	1.23	0.11	**0.03**	0.94	0.43	0.11	**0.03**	0.54	0.22	0.13	**0.01**	0.04	0.38	0.00	0.92	0.82	0.30	0.14	**0.01**	0.30	0.21	0.07	0.16
Violence	−3.13	4.32	−0.05	0.47	−0.80	1.52	−0.04	0.60	−0.52	0.78	−0.05	0.50	−0.63	1.35	−0.03	0.64	−0.61	1.07	−0.04	0.57	−0.58	0.75	−0.06	0.44
Low trauma and contextual stress (ref)	---	---	---	---	---	---	---	---	---	---	---	---	---	---	---	---	---	---	---	---	---	---	---	---
Race/ethnicity																								
Black	−0.93	0.87	−0.05	0.29	−0.09	0.30	−0.01	0.77	0.26	0.16	0.09	0.09	−0.31	0.27	−0.05	0.26	−0.87	0.21	−0.18	**<0.001**	0.07	0.15	0.02	0.62
Hispanic/Latina	−1.58	1.05	−0.07	0.13	−0.63	0.37	−0.07	0.08	−0.26	0.19	0.17	0.17	−0.14	0.32	−0.02	0.66	−0.80	0.26	−0.14	**0.002**	0.25	0.18	0.06	0.16
White (ref)	---	---	---	---	---	---	---	---	---	---	---	---	---	---	---	---	---	---	---	---	---	---	---	---
Class × race/ethnicity omnibus test	*F* (6, 1554) = 2.24, *p* = 0.04				*F* (6, 1554) = 2.31, *p* = 0.03				*F* (6, 1554) = 1.84, *p* = 0.09				*F* (6, 1554) = 2.32, *p* = 0.03				*F* (6, 1554) = 1.65, *p* = 0.13				*F* (6, 1554) = 1.28, *p* = 0.27			
	*b*	s.e.	*β*	*p*	*b*	s.e.	*β*	*p*	*b*	s.e.	*β*	*p*	*b*	s.e.	*β*	*p*	*b*	s.e.	*β*	*p*	*b*	s.e.	*β*	*p*
High Contextual Stress × Black	−1.44	1.96	−0.04	0.46	0.02	0.68	0.00	0.97	0.01	0.35	0.00	0.97	−0.35	0.61	−0.03	0.57	−0.48	0.48	−0.06	0.32	−0.65	0.34	−0.12	0.06
Injury/Illness × Black	1.53	1.48	0.05	0.31	0.48	0.52	0.04	0.36	−0.25	0.26	−0.05	0.35	1.03	0.46	0.11	**0.02**	0.14	0.36	0.02	0.69	0.12	0.26	0.02	0.63
Violence Exposure × Black	10.73	4.66	0.16	**0.02**	3.41	1.63	0.14	**0.04**	1.48	0.84	0.12	0.08	2.14	1.44	0.10	0.14	2.38	1.15	0.15	**0.04**	1.32	0.81	0.11	0.10
High Contextual Stress × Latina	1.93	2.20	0.04	0.38	1.44	0.77	0.08	*0*.*06*	0.63	0.39	0.07	0.11	0.61	0.68	0.04	0.37	−0.33	0.54	−0.03	0.55	−0.43	0.38	−0.05	0.26
Injury/Illness × Latina	−0.24	1.80	−0.01	0.90	0.19	0.63	0.01	0.76	−0.34	0.32	−0.40	0.30	0.31	0.56	0.02	0.57	−0.41	0.44	−0.04	0.35	0.01	0.31	0.00	0.97
Violence Exposure × Latina	9.55	6.13	0.05	0.12	4.43	2.14	0.07	**0.04**	1.81	1.10	0.06	0.10	1.52	1.90	0.03	0.42	0.41	1.51	0.01	0.79	1.38	1.06	0.05	0.19

PTSD, posttraumatic stress disorder.

*Note*: All models adjusted for mother's age, education, region, and poverty level. Low Trauma/Contextual Stress class and White race/ethnicity as reference groups. Significant *p* values ≤ 0.05 in **bold**. *p* values ≤ 0.10 are italicized, although these values did not reach statistical significance.

For reexperiencing, differences in symptom severity between Black and White women were greater within the *Violence Exposure* class than in the *Low Trauma & Contextual Stress* class (*b* = 3.41, *p* = 0.04). Likewise, differences in symptom severity between Hispanic/Latina and White women were greater within the *Violence Exposure* class than in the *Low Trauma & Contextual Stress* class (*b* = 4.43 *p* = 0.04). Finally, there was a greater difference in reexperiencing severity scores between Black and Hispanic/Latina women in the *High Contextual Stress* class than in the *Low Trauma & Contextual* Stress class (*b* = −1.42, *p* = 0.01). Examination of within class contrasts revealed that Black women in the *Violence Exposure* class had higher reexperiencing symptom severity than White women (online Supplementary Table S5). Although not reaching statistical significance, Black women within the *Injury*/*Illness* class had higher reexperiencing symptom severity than Hispanic/Latina women. While also not reaching statistical significance, Hispanic/Latina women within the *Violence Exposure* class had greater reexperiencing symptom severity than White women. Likewise, Hispanic/Latina women within the *High Contextual Stress* class had greater reexperiencing symptom severity than Black women.

Finally, within the numbing symptom dimension, there was a greater difference in severity scores between Black and White women in the *Injury*/*Illness* class than in the *Low Trauma & Contextual Stress* (*b* = 1.03, *p* = 0.02). Additionally, there was a greater difference in numbing severity scores between Black and Hispanic/Latina women in the *High Contextual Stress* class than in the *Low Trauma & Contextual* Stress class (*b* = −0.96, *p* = 0.05). Within class contrasts revealed that, within the *Injury*/*Illness* class, Black women had greater symptom severity than White women, although not statistically significant (online Supplementary Table S5). Within the *High Contextual Stress* class, Latina/Hispanic women had greater numbing symptom severity than Black women.

## Discussion

The current study used a person-centered approach to examine trauma and contextual stress and their associations with PTSD symptoms in an ethnically and racially diverse sample of low-income postpartum women. Several key findings emerged. First, women showed high levels of PTE and contextual stress exposure in a relatively short time frame (i.e. past year) within the perinatal period. Second, using LCA, we identified four classes based on exposures women experienced: *High Contextual Stress, Injury*/*Illness, Violence Exposure, and Low Trauma & Contextual Stress*. Third, patterning of exposure to trauma and contextual stressors, but not race/ethnicity alone, was robustly and differentially related to PTSD symptoms; compared to experiencing lower levels of trauma and contextual stress, membership in any of the other three classes was associated with greater symptom severity across nearly all PTSD symptom measures. Fourth, constellations of exposures were differentially linked to PTSD symptoms across racial and ethnic groups. Moreover, considering underlying dimensions of PTSD revealed additional associations not captured by total symptom severity, particularly for reexperiencing and numbing symptoms.

The high levels of trauma exposures evidenced in our sample, particularly IPV (35%), is notable and consistent with research documenting disproportionately high violence histories among low-income women both proximally and across the lifetime (Golin et al., 2017; Velonis et al., 2017). In our sample, two distinct groups emerged: one characterized by high levels of trauma in the form of injury or illness and another characterized by high levels of violence exposure, including threats of physical harm and IPV. These trauma exposures occurred alongside moderate levels of contextual stress exposure. A third group was characterized by high levels of contextual stressors, but lower levels of trauma exposure. The fourth and largest class was characterized by relatively lower levels of trauma and contextual stress exposure, though these experiences were still prevalent. These findings align with the few studies using LCA to examine patterning of trauma exposure, typically showing 3–4 class models to best fit the data, and at least one class characterized by high violence exposure and one class characterized by low trauma exposure overall (O'Donnell et al., 2017). The inclusion of contextual stressors and IPV in our study revealed a gradient pattern across classes, suggesting that these exposures co-occurred at varying levels of severity for all women in this diverse, predominantly low-income sample.

Class membership emerged as an important predictor of PTSD symptoms. Different classes of trauma and contextual stress exposure were significantly associated with PTSD symptoms at the 6-month postpartum assessment – both total symptom severity and the dimensions of the dysphoric arousal model – with the largest effects typically seen for *Violence Exposure* and *High Contextual Stress*. Race/ethnicity on its own, however, was not robustly associated with PTSD symptoms, except for greater avoidance symptom severity in Black *v.* Hispanic/Latina women, and greater dysphoric arousal symptom severity in White *v.* Black and Hispanic/Latina women. These findings are consistent with previous work in this sample (Thomas et al., 2021a).

With the inclusion of Class × Race/ethnicity interactions, more nuanced findings emerged, showing that class membership was differentially associated with PTSD symptoms as a function of race/ethnicity, but particularly for certain PTSD dimensions. For example, *Violence Exposure* was particularly impactful in terms of severity of reexperiencing symptoms for Black compared to White women, although no differences in symptom severity were found between violence-exposed Black and Hispanic/Latina women. Importantly, the *Violence Exposure* class had relatively high levels of discrimination, potentially adding to or acting synergistically with experiences of violence in Black women. Research has shown that racism-related stressors may confer susceptibility to trauma-related disorders by altering threat-related neurophysiology in Black Americans (Webb, Carter, Ressler, Fani, & Harnett, 2024). Detecting these associations for reexperiencing symptoms in particular – core symptoms of PTSD characterized by intrusive thoughts or memories about the trauma and related reactivity – after exposure to interpersonal violence is notable, as these are often highly personally threatening PTEs. Indeed, of the various PTSD symptoms, intrusions have been proposed to serve as warning signals for potential danger, thereby triggering a sense of current threat (Ehlers, Hackmann, & Michael, 2004). Furthermore, these types of symptoms may be especially relevant for adverse maternal-infant health during a period already sensitive to stress and physiological changes (Valsamakis, Chrousos, & Mastorakos, 2019).

Differences were also observed between Hispanic/Latina and Black women, such that within the *High Contextual Stress* class, Hispanic/Latina women had greater numbing symptom severity. It is possible that high levels of numbing symptoms in Hispanic/Latina mothers under conditions of high contextual stress may indicate overwhelming or chronic stressors (e.g. financial stress) as opposed to one-time or acute traumatic exposures. Emotional distancing and suppression may be ways to cope with these types of prolonged exposures; numbing is characterized by restricted affect and interpersonal detachment (Wisco, Pugach, & Nomamiukor, 2020), with negative implications for mother-infant interactions and family dynamics. Previous research in this sample strongly linked numbing to maternal stress, parenting stress, and poor interpersonal functioning (Thomas et al., 2021b). Lack of significant interactions for other symptom dimensions, particularly dysphoric arousal and anxious arousal, may be an artifact of the postpartum period, where elements of these dimensions may overlap with characteristics of being a new mother, such as sleep and concentration difficulties, or vigilance for an infant's needs (Christian, Carroll, Teti, & Hall, 2019; Puri, Richard, & Galea, 2023). Therefore, these symptom dimensions may be less influenced by past exposures during a period of postpartum adjustment. However, future research is needed to test these hypotheses. Finally, cultural factors may influence manifestation of PTSD symptoms, including numbing, due to cultural norms regarding emotional expression and interpretations of distress, although research on this topic is limited and should be explored further (Ceja, Yalch, & Maguen, 2022; Ford, Grasso, Elhai, & Courtois, 2015).

This study has several strengths and limitations. First, this study utilizes a unique community-based sample of postpartum women, providing an opportunity to examine traumatic stress in a low-income, racially and ethnically diverse sample. While we were unable to capture all possible relevant experiences of trauma and contextual stress (e.g. childhood/lifetime trauma, pregnancy-specific trauma, sexual violence), this is one of the few studies to examine data-driven constellations of various past-year exposures in this population. Still, given the exploratory nature of LCAs, findings from this study are preliminary, and further research is needed to determine the class structure that most accurately or meaningfully characterizes experiences of trauma and contextual stress exposure. Future studies should also expand on this research by considering additional relevant exposures and time frames. Moreover, building on this person-centered approach by examining cumulative burden and severity of exposures, which can be done using extensions of LCA (e.g. latent profile analysis), is an important next step, as these factors are critical in understanding the overall impact on PTSD symptomatology. Comparisons across all race/ethnic groups allowed for a more nuanced understanding of experiences and their consequences. More research is needed to replicate these findings, which may have limited detection of all possible interactions. Still, as emphasized by guidelines for best practices for examining race/ethnicity in health research (Flanagin, Frey, & Christiansen, 2021; Ross, Hart-Johnson, Santen, & Zaidi, 2020), results showed that race/ethnicity on its own is not explanatory for PTSD; rather, it is the associated experiences that can shape observed disparities in PTSD symptom severity. To this end, the comprehensive person-centered approach to capturing exposures used in this study is an important, yet underused, tool for understanding trauma, the context in which it occurs, and its implications for psychopathology.

Understanding the context of trauma is crucial, as many women from marginalized communities face compounded stressors. Current DSM criteria for traumatic experiences focus on discrete, severe events, often excluding contextual stressors that shape traumatic experiences (Gradus & Galea, 2023). Given public health priorities to address population health beyond the most severe, clinical cases, there has been a push to redefine trauma to include contexts that can shape traumatic reactions (e.g. poverty) and events traditionally not considered traumatic but linked to posttraumatic psychological distress (e.g. racism; Gradus & Galea, 2022; Gradus & Galea, 2023). Findings from this study support this broader approach, advocating for a more holistic understanding of trauma and its impact during critical time frames.

## Conclusions

High rates of trauma and contextual stress exposure, along with racial and ethnic differences in PTSD symptoms, highlight the perinatal period as a window of opportunity for screening and intervention. Increased engagement with the healthcare system during this time facilitates early identification of women at risk for posttraumatic psychopathology and other common mental health disorders, with the potential to improve long-term well-being for both mother and child. Considering context when addressing trauma and mental health during the perinatal period can also help reduce pervasive disparities in perinatal outcomes. Racial/ethnic minority and low-income mothers typically enter the perinatal period facing multiple stressors within SDoH domains. Targeted support and culturally sensitive trauma-informed care that consider and screen for these stressors could mitigate some of the associated psychological burden, and include collaborative care models, psychiatric resources for treating clinicians and their pregnant or postpartum patients, and collaboration with community organizations to provide wraparound services (Wisner et al., 2024). Structurally, this approach could foster a more equitable healthcare environment, allowing all women to have a thriving pregnancy, improving the health of mother and baby.

## Supporting information

Kofman et al. supplementary material Kofman et al. supplementary material

## Supplementary material

The supplementary material for this article can be found at https://doi.org/10.1017/S0033291724002915.
