# Supplementary material for: Trauma exposure, contextual stressors, and PTSD symptoms: patterns in racially and ethnically diverse, low-income postpartum women
Source: Psychol Med. 2025 Jan 14;54(16):4747–58. doi: 10.1017/S0033291724002915 (PMC11779552; doi:10.1017/S0033291724002915)
Supplement: Kofman et al. supplementary material [file S0033291724002915sup001.docx]

Online Supplemental Material

Supplemental Table 1. Model fit indices for the LCA model with 1-6 classes

| Classes | par | LL | BIC | aBIC | CIAC | BLRT | VLMR |
| --- | --- | --- | --- | --- | --- | --- | --- |
| Class-1 | 11 | −6,760 | 13,601 | 13,566 | 13,612 | – | – |
| Class-2 | 23 | −6,619 | 13,408 | 13,335 | 13,431 | <.001 | .01 |
| Class-3 | 35 | −6,577 | 13,411 | 13,300 | 13,446 | <.001 | <.001 |
| Class-4 | 47 | −6,555 | 13,456 | 13,307 | 13,503 | <.001 | .43 |
| Class-5 | 59 | −6,538 | 13,510 | 13,323 | 13,569 | <.001 | .31 |
| Class-6 | 71 | −6,527 | 13,576 | 13,350 | 13,647 | .43 | .06 |
| *Note.*aBIC=sample size adjusted BIC; BIC=bayesian information criterion; BLRT=bootstrapped likelihood ratio test *p*-value; CAIC=consistent Akaike information criterion; LL=log likelihood; Par=parameters; VLMR=Vuong-Lo-Mendell-Rubin adjusted likelihood ratio test *p*-value. | | | | | | | |

| Supplemental Table 2. Model classification proportions and accuracy | | | | | |
| --- | --- | --- | --- | --- | --- |
| k-class solution | k-class | k-class proportions | mcaP_k_ | AvePP_k_ | Entropy |
| 2-Class | Class 1 | .13 | .10 | .81 | .63 |
|  | Class 2 | .86 | .90 | .94 |  |
| 3-Class | Class 1 | .03 | .03 | .90 | .57 |
|  | Class 2 | .45 | .47 | .80 |  |
|  | Class 3 | .52 | .51 | .73 |  |
| 4-Class | Class 1 | .21 | .17 | .67 | .58 |
|  | Class 2 | .25 | .23 | .70 |  |
|  | Class 3 | .03 | .03 | .89 |  |
|  | Class 4 | .51 | .57 | .76 |  |
| 5-Class | Class 1 | .51 | .57 | .77 | .60 |
|  | Class 2 | .03 | .03 | .87 |  |
|  | Class 3 | .25 | .23 | .78 |  |
|  | Class 4 | .01 | .01 | .89 |  |
|  | Class 5 | .21 | .17 | .70 |  |
| *Note*.  AvePP_k_ = average posterior probability of correct classification; mcaP_k_ = modal class assignment. | | | | | |

| Supplemental Table 3. Sociodemographics by class (n=1577) | | | | | |
| --- | --- | --- | --- | --- | --- |
| Variable | High Contextual Stress  (n=265) | Injury/Illness (n=353) | Violence Exposure (n=45) | Low Trauma/  Contextual Stress  (n=914) | *p* |
| Age | 25.17 (5.28) | 25.07 (5.55) | 25.57 (5.21) | 26.28 (5.89) | **.002** |
| Study site |  |  |  |  | **<.001** |
| Baltimore | 56 (21.13) | 69 (19.55) | 14 (31.11) | 215 (23.52) |  |
| Chicago | 48 (18.11) | 82 (23.23) | 4 (8.89) | 270 (29.54) |  |
| Los Angeles | 35 (13.21) | 40 (11.33) | 8 (17.78) | 99 (10.83) |  |
| North Carolina | 56 (21.13) | 87 (24.65) | 8 (17.78) | 175 (19.15) |  |
| Washington, D.C. | 70 (26.42) | 75 (21.25) | 11 (24.44) | 155 (16.96) |  |
| Poverty |  |  |  |  | **<.001** |
| ≤ 100% FPL | 149 (56.23) | 153 (43.34) | 24 (53.33) | 353 (38.62) |  |
| 100-200% FPL | 71 (26.79) | 100 (28.33) | 6 (13.33) | 253 (27.68) |  |
| >200% FPL | 45 (16.98) | 100 (28.33) | 15 (33.33) | 308 (33.70) |  |
| Education |  |  |  |  | **<.001** |
| Less than high school | 54 (20.38) | 52 (14.73) | 7 (15.56) | 167 (18.27) |  |
| High school, GED certificate | 125 (47.17) | 146 (41.36) | 20 (44.44) | 378 (41.36) |  |
| Some college | 64 (24.15) | 103 (29.18) | 15 (33.33) | 191 (20.90) |  |
| 4-year degree or higher | 18 (6.79) | 51 (14.45) | 3 (6.67) | 169 (18.49) |  |
| Other, no information | 4 (1.51) | 1 (0.28) | -- | 9 (0.98) |  |
| Race |  |  |  |  | **<.001** |
| Black | 161 (60.75) | 197 (55.81) | 35 (77.78) | 457 (50.0) |  |
| White | 35 (13.21) | 81 (22.95) | 5 (11.11) | 253 (27.68) |  |
| Hispanic/Latina | 69 (26.04) | 75 (21.25) | 5 (11.11) | 204 (22.32) |  |
| *Note. N* (%) for categorical variables; *M* (SD) for continuous variables. Significant *p*-values *≤* .05 in **bold**. FPL=federal poverty level. | | | | | |

| Supplemental Table 4. Class membership $\times$ race/ethnicity interactions and associations with PTSD symptom dimensions | | | | | | | | | | | | | | | | | | | | | | | | |
| --- | --- | --- | --- | --- | --- | --- | --- | --- | --- | --- | --- | --- | --- | --- | --- | --- | --- | --- | --- | --- | --- | --- | --- | --- |
|  | **Total PTSD** | | | | **Reexperiencing** | | | | **Avoidance** | | | | **Numbing** | | | | **Dysphoric Arousal** | | | | **Anxious Arousal** | | | |
|  | *F (*22,1554) = 9.02,  *p* <.001 | | | | *F (*22,1554) = 8.67,  *p* <.001 | | | | *F (*22,1554) = 7.36,  *p* <.001 | | | | *F (*22,1554) = 7.27,  *p* <.001 | | | | *F (*22,1554) = 5.02,  *p* <.001 | | | | *F (*22,1554) = 5.07,  *p* <.001 | | | |
|  | *b* | SE | β | *p* | *b* | SE | β | *p* | *b* | SE | β | *p* | *b* | SE | β | *p* | *b* | SE | β | *p* | *b* | SE | β | *p* |
|  |  |  |  |  |  |  |  |  |  |  |  |  |  |  |  |  |  |  |  |  |  |  |  |  |
| **Class** |  |  |  |  |  |  |  |  |  |  |  |  |  |  |  |  |  |  |  |  |  |  |  |  |
| High Stress | 6.53 | 1.34 | 0.24 | **<.001** | 2.39 | 0.47 | 0.25 | **<.001** | 1.00 | .24 | .21 | **<.001** | 1.97 | 0.42 | 0.24 | **<.001** | 0.84 | 0.33 | 0.13 | **.01** | 0.33 | 0.23 | 0.07 | .16 |
| Injury & Illness | 2.40 | 1.31 | 0.10 | .17 | 1.13 | 0.46 | 0.13 | **.01** | 0.20 | .24 | **.05** | **.39** | 0.35 | 0.41 | 0.05 | .39 | 0.41 | 0.32 | 0.07 | .21 | 0.31 | 0.23 | 0.08 | .17 |
| Violence | 6.42 | 4.36 | 0.11 | .14 | 3.63 | 1.52 | 0.17 | **.02** | 1.29 | .78 | .12 | *.10* | 0.89 | 1.35 | 0.05 | .51 | -0.20 | 1.08 | -0.01 | .85 | 0.81 | 0.76 | 0.08 | .29 |
| Low Trauma & Contextual Stress (ref) | --- | --- | --- | --- | --- | --- | --- | --- | --- | --- | --- | --- | --- | --- | --- | --- | --- | --- | --- | --- | --- | --- | --- | --- |
|  |  |  |  |  |  |  |  |  |  |  |  |  |  |  |  |  |  |  |  |  |  |  |  |  |
| **Race/ethnicity** |  |  |  |  |  |  |  |  |  |  |  |  |  |  |  |  |  |  |  |  |  |  |  |  |
| Black | 0.66 | 0.96 | **0.03** | .49 | 0.54 | 0.34 | .08 | .10 | 0.52 | 0.17 | .15 | **.002** | -0.17 | 0.30 | -0.03 | .58 | -0.06 | 0.24 | -0.01 | .79 | -0.18 | 0.17 | -0.05 | .28 |
| White | 1.58 | 1.05 | 0.07 | .13 | 0.63 | 0.37 | 0.07 | .08 | 0.26 | 0.19 | -0.17 | .17 | 0.14 | 0.32 | 0.02 | .66 | 0.80 | 0.26 | 0.14 | **.002** | -0.25 | 0.18 | -0.06 | .16 |
| Hispanic/Latina (ref) | --- | --- | --- | --- | --- | --- | --- | --- | --- | --- | --- | --- | --- | --- | --- | --- | --- | --- | --- | --- | --- | --- | --- | --- |
|  |  |  |  |  |  |  |  |  |  |  |  |  |  |  |  |  |  |  |  |  |  |  |  |  |
| **Class** $\boldsymbol{\times}$ **Race/ethnicity omnibus test** | *F* (6, 1554) = 2.24,  *p* = .04 | | | | *F* (6, 1554) = 2.31,  *p* =.03 | | | | *F* (6, 1554) = 1.84,  *p* = .09 | | | | *F* (6, 1554) = 2.32,  *p* = .03 | | | | *F* (6, 1554) = 1.65,  *p* = .13 | | | | *F* (6, 1554) = 1.28,  *p* = .27 | | | |
|  | *b* | SE | β | *p* | *b* | SE | β | *p* | *b* | SE | β | *p* | *b* | SE | β | *p* | *b* | SE | β | *p* | *b* | SE | β | *p* |
| High Contextual Stress $\times$ White | -1.93 | 2.20 | -0.03 | .38 | -1.44 | 0.77 | -0.08 | *.06* | -0.63 | 0.39 | -0.07 | .11 | -0.61 | 0.68 | -0.04 | .37 | 0.33 | 0.54 | 0.03 | .55 | 0.43 | 0.38 | 0.05 | .26 |
| Injury/Illness $\times$ White | 0.24 | 1.80 | 0.01 | .90 | -0.19 | 0.63 | -0.01 | .76 | 0.34 | 0.32 | 0.40 | .30 | -0.31 | 0.56 | -0.02 | .57 | 0.41 | 0.44 | 0.04 | .35 | -0.01 | 0.31 | -0.00 | .97 |
| Violence Exposure $\times$ White | -9.55 | 6.13 | -0.05 | .12 | -4.43 | 2.14 | -0.07 | **.04** | -1.81 | 1.10 | -0.06 | .10 | -1.52 | 1.90 | -0.03 | .42 | -0.41 | 1.51 | -0.01 | .79 | -1.38 | 1.06 | -0.05 | .19 |
| High Contextual Stress $\times$Black | -3.37 | 1.60 | -0.10 | **.04** | -1.42 | 0.56 | -0.12 | **.01** | -0.62 | 0.29 | -0.10 | .03 | -0.96 | 0.50 | -0.09 | **.05** | -0.15 | 0.39 | -0.02 | .70 | -0.22 | 0.28 | -0.04 | .43 |
| Injury/Illness$\times$ Black | 1.76 | 1.55 | 0.06 | .26 | 0.29 | 0.54 | 0.027 | .59 | 0.09 | 0.28 | 0.02 | .75 | 0.72 | 0.48 | -0.08 | .14 | 0.56 | 0.38 | 0.08 | .14 | 0.11 | 0.27 | 0.02 | .67 |
| Violence Exposure $\times$Black | 1.18 | 4.67 | 0.02 | .80 | -1.02 | 1.63 | -0.04 | .53 | -0.34 | 0.84 | -0.03 | .69 | 0.61 | 1.45 | 0.03 | .67 | 1.98 | 1.15 | 0.12 | .09 | -0.06 | 0.81 | -0.01 | .94 |

*Note*. All models adjusted for mother’s age, education, region, and poverty level. Low Trauma/Contextual Stress class and Hispanic/Latina race/ethnicity as reference groups. Significant *p*-values *≤* .05 in **bold.** P-values *≤* .10 are italicized, although these values did not reach statistical significance. PTSD=posttraumatic stress disorder.

| Supplemental Table 5. Contrast of marginal linear predictions | | | | | | | | | | | | |
| --- | --- | --- | --- | --- | --- | --- | --- | --- | --- | --- | --- | --- |
|  | **Total PTSD** | | | | **Reexperiencing** | | | | **Numbing** | | | |
|  | Contrast | SE | *t* | *p* | Contrast | SE | *t* | *p* | Contrast | SE | *t* | *p* |
|  |  |  |  |  |  |  |  |  |  |  |  |  |
| High Contextual Stress $\times$ Black (vs. White) | -2.37 | 1.83 | -1.30 | .20 | -0.06 | 0.64 | -0.10 | .92 | -0.66 | 0.57 | -1.16 | .25 |
| Injury/Illness $\times$ Black (vs. White) | 0.60 | 1.33 | 0.45 | .65 | 0.39 | 0.46 | 0.84 | .40 | 0.72 | 0.41 | 1.76 | *.08* |
| Violence Exposure $\times$ Black (vs. White) | 9.80 | 4.61 | 2.12 | **.03** | 3.32 | 1.61 | 2.06 | **.04** | 1.83 | 1.43 | 1.28 | .20 |
| Low Trauma & Contextual Stress $\times$ Black (vs. White) | -0.93 | 0.87 | -1.06 | .29 | -0.09 | 0.30 | -0.29 | .77 | -0.31 | 0.27 | -1.14 | .26 |
|  |  |  |  |  |  |  |  |  |  |  |  |  |
| High Contextual Stress $\times$ Hispanic/Latina (vs. White) | 0.35 | 2.05 | 0.17 | .87 | 0.81 | 0.71 | 1.13 | .26 | 0.47 | 0.63 | 0.75 | .46 |
| Injury/Illness $\times$ Hispanic/Latina (vs. White) | -1.82 | 1.62 | -1.12 | .26 | -0.44 | 0.57 | -0.78 | .43 | 0.17 | 0.50 | 0.35 | .73 |
| Violence Exposure $\times$ Hispanic/Latina (vs. White) | 7.97 | 6.09 | 1.31 | .19 | 3.80 | 2.13 | 1.78 | *.08* | 1.38 | 1.89 | 0.73 | .46 |
| Low Trauma & Contextual Stress $\times$ Hispanic/Latina (vs. White) | -1.58 | 1.05 | -1.51 | .13 | -0.63 | 0.37 | -1.73 | *.08* | -0.14 | 0.32 | -0.43 | .66 |
|  |  |  |  |  |  |  |  |  |  |  |  |  |
| High Contextual Stress $\times$Black (vs. Hispanic/Latina) | -2.72 | 1.44 | -1.89 | *.06* | -0.87 | 0.50 | -1.74 | *.08* | -1.13 | 0.44 | -2.54 | **.01** |
| Injury/Illness$\times$ Black (vs. Hispanic/Latina) | 2.42 | 1.37 | 1.77 | *.08* | 0.83 | 0.48 | 1.74 | *.08* | 0.55 | 0.43 | 1.29 | .20 |
| Violence Exposure $\times$Black (vs. Hispanic/Latina) | 1.83 | 4.63 | 0.40 | .69 | -0.47 | 1.62 | -0.29 | .77 | 0.45 | 1.44 | 0.31 | .76 |
| Low Trauma & Contextual Stress $\times$ Black (vs. Hispanic/Latina) | 0.66 | 0.96 | 0.69 | .49 | 0.54 | 0.34 | 1.62 | .11 | -0.17 | 0.30 | -0.56 | .58 |

*Note.* Coefficient indicates discrete change from base level (reference group). Significant *p*-values *≤* .05 in **bold.** P-values *≤* .10 are italicized, although these values did not reach statistical significance. PTSD=posttraumatic stress disorder.
